# Cost-Effectiveness of Pantoprazole to Prevent Upper Gastrointestinal Bleeding in Mechanically Ventilated Patients

**DOI:** 10.1001/jamanetworkopen.2025.52771

**Published:** 2025-12-01

**Authors:** Feng Xie, Yifan Yao, Yue Ma, Brittany Humphries, Vincent I. Lau, Robert Fowler, Bram Rochwerg, Diane Heels-Ansdell, Nicole Zytaruk, Jeffrey F. Barletta, Salmaan Kanji, Yaseen M. Arabi, Daniel W. Johnson, David Williamson, John C. Marshall, Adam Deane, John Myburgh, Anna Geagea, Alex Poole, Patrick Archambault, Kosar Khwaja, Marlies Ostermann, Lisa Burry, Gordon H. Guyatt, Deborah J. Cook

**Affiliations:** 1 Department of Health Research Methods, Evidence, and Impact, McMaster University, Hamilton, Ontario, Canada; 2 Centre for Health Economics and Policy Analysis, McMaster University, Hamilton, Ontario, Canada; 3 Department of Medicine, McMaster University, Hamilton, Ontario, Canada; 4 Department of Critical Care Medicine, Faculty of Medicine and Dentistry, University of Alberta, Edmonton, Alberta, Canada; 5 Interdepartmental Division of Critical Care, University of Toronto, Toronto, Ontario, Canada; 6 Centre for Health Economics and Policy Analysis, McMaster University, Hamilton, Canada; 7 Department of Pharmacy Practice, Midwestern University, College of Pharmacy-Glendale Campus, Glendale, Arizona; 8 The Ottawa Hospital Research Institute, University of Ottawa, Ottawa, Ontario, Canada; 9 King Abdullah International Medical Research Center, King Saud Bin Abdulaziz University for Health Sciences, Riyadh, Saudi Arabia; 10 Critical Care Division, Department of Anesthesiology, University of Nebraska Medical Center, Omaha; 11 Faculty of Pharmacy, Université de Montréal, Sacré-Coeur Hospital in Montréal, Montreal, Quebec, Canada; 12 Interdepartmental Division of Critical Care, University of Toronto, Toronto, Ontario, Canada; 13 University of Melbourne, Melbourne, Victoria, Australia; 14 The George Institute for Global Health, Faculty of Medicine and Health, University of New South Wales, Sydney, New South Wales, Australia; 15 Department of Critical Care, North York General Hospital, Toronto, Ontario, Canada; 16 University of Adelaide, Adelaide, South Australia, Australia; 17 Université Laval, Québec City, Quebec, Canada; 18 Departments of Critical Care and Medicine, McGill University, Montréal, Quebec, Canada; 19 Guys’ and St Thomas Hospital, London, England; 20 Department of Pharmacy, Sinai Health, Toronto, Ontario, Canada

## Abstract

**Importance:**

Pantoprazole reduces clinically important upper gastrointestinal bleeding in critically ill patients. However, the cost-effectiveness of this strategy is unclear.

**Objective:**

To assess the cost-effectiveness of daily intravenous pantoprazole vs no pantoprazole for preventing upper gastrointestinal bleeding in mechanically ventilated patients.

**Design, Setting, and Participants:**

This prospective health economic evaluation was conducted alongside the Reevaluating the Inhibition of Stress Erosions (REVISE) trial over a time horizon of intensive care unit (ICU) admission to hospital discharge or death from a public health care payer’s perspective. The REVISE trial included critically ill adults from Canada, Australia, the US, England, Saudi Arabia, Brazil, Kuwait, and Pakistan receiving invasive ventilation.

**Interventions:**

Daily intravenous pantoprazole (40 mg) or placebo (0.9% sodium chloride).

**Main Outcomes and Measures:**

The primary outcome was the incremental cost per clinically important upper gastrointestinal bleed prevented. The base-case analysis included all study site–specific resource utilization. Canadian costs were applied to measured resource uses across sites. Sensitivity analyses were conducted across cost ranges and using US-based cost estimates. Uncertainty was assessed using nonparametric bootstrapping simulations. All costs are presented in 2025 US dollars.

**Results:**

4821 invasively ventilated critically ill patients (mean [SD] age, 58.2 [16.4] years; 1752 female [36.3%]) were enrolled from 68 ICUs. For pantoprazole, the mean (SD) stay was 12.4 (11.7) days in the ICU and an additional 14.8 (28.0) days in the hospital, as compared with 13.3 (13.3) days in the ICU and 16.5 (42.9) days in the hospital for no pantoprazole. Mean (SD) total per-patient costs were $60 466 ($58 546) for pantoprazole vs $65 423 ($75 661) for no pantoprazole. The incremental cost per patient was −$4957 (95% CI, −$8777 to −$1136). In a sensitivity analysis, US costs were applied for pantoprazole, bleeding, and ICU and hospital stay to all patients; mean (SD) total per-patient costs were $130 179 ($123 456) for pantoprazole vs $140 770 ($153 195) for no pantoprazole (incremental cost: −$10 591; 95% CI, −$18 448 to $−2735). When excluding top 10% of patients in terms of ICU days, ward days, and total costs, the incremental costs were −$1151, −$3388, and −$1356, respectively. In 99% of simulations, the strategy of using pantoprazole was more effective and less costly than no pantoprazole.

**Conclusions and Relevance:**

In this economic evaluation, daily pantoprazole for invasively mechanically ventilated patients was less costly and more effective than care without pantoprazole, indicating both clinical benefits and economic value for the health care system.

## Key Points

**Question:**

Is daily intravenous pantoprazole cost-effective in preventing upper gastrointestinal bleeding in mechanically ventilated patients compared with no pantoprazole?

**Findings:**

This economic evaluation including 4821 patients from the Reevaluating the Inhibition of Stress Erosions trial found that prophylactic pantoprazole was associated with a significantly lower rate of clinically important upper gastrointestinal bleeding and a lower mean total cost per patient compared with no pantoprazole.

**Meaning:**

This economic evaluation found that prescribing daily pantoprazole for invasively mechanically ventilated patients was less costly and more effective than care without pantoprazole, indicating both clinical benefits and economic value for the health care system.

## Teaser

This economic evaluation examines cost-effectiveness of daily intravenous pantoprazole vs no pantoprazole for preventing upper gastrointestinal bleeding in critically ill, mechanically ventilated patients from the Reevaluating the Inhibition of Stress Erosions trial.

## Introduction

Proton pump inhibitors (PPIs) are routinely administered to critically ill patients to prevent upper gastrointestinal bleeding from stress-induced ulceration.^1^ Two recent large international randomized trials evaluated acid suppression with pantoprazole in this population. The Stress Ulcer Prophylaxis in the Intensive Care Unit (SUP-ICU) trial^2^ enrolled 3298 patients in the ICU and showed that patients receiving pantoprazole had reduced clinically important gastrointestinal bleeding compared with placebo and similar 90-day mortality. The Re-Evaluating the Inhibition of Stress Erosions (REVISE) trial^3^ enrolled 4821 invasively ventilated critically ill patients and found a lower risk of clinically important upper gastrointestinal bleeding in patients receiving pantoprazole compared to placebo, with a similar risk of death at 90 days.

Economic outcomes are important in critical care research.^4^ Cost analyses comparing individual agents used for stress ulcer prophylaxis have been performed through simulation modeling, but the cost-effectiveness of stress ulcer prophylaxis with pantoprazole remains unclear.^5,6,7^ This study aimed to evaluate the cost-effectiveness of stress ulcer prophylaxis alongside the REVISE trial.

## Methods

The objective of this health economic evaluation of the REVISE trial (E-REVISE) was to estimate the cost-effectiveness of prophylactic pantoprazole compared with no pantoprazole to prevent clinically important upper gastrointestinal bleeding among invasively ventilated patients. REVISE was an investigator-initiated, international, randomized, blinded, controlled trial (NCT03374800). Patients aged 18 years or older who were expected to remain invasively ventilated beyond the calendar day after randomization were randomized to either placebo (0.9% sodium chloride) or intravenous pantoprazole (40 mg daily).^3^ Randomization was stratified by study center and prehospital acid suppression. Care was otherwise at the discretion of the treating team. The primary efficacy outcome of the trial was clinically important upper gastrointestinal bleeding within 90 days, and the primary safety outcome was 90-day mortality. Secondary trial outcomes included ventilator-associated pneumonia, *Clostridioides difficile* infection, and patient important upper gastrointestinal bleeding. The REVISE protocol,^8^ statistical analysis plan,^9^ and results^3^ were previously published. This health economic evaluation was based on a separate published protocol and statistical analysis plan,^10^ conducted alongside the REVISE trial following global guidance from the International Society for Pharmacoeconomics and Outcomes Research (ISPOR) reporting guideline^11^ and guidance from Canada’s Drug Agency.^12^ This analysis was reported according to the Consolidated Health Economic Evaluation Reporting Standards (CHEERS) reporting guideline.^13^

The primary outcome of this economic evaluation was the incremental cost per clinically important upper gastrointestinal bleed prevented. E-REVISE was conducted from a Canadian public health care payer’s perspective over a time horizon of ICU admission to hospital discharge or death. The base-case analysis of E-REVISE included all patients enrolled in the trial.

The REVISE trial was approved by relevant human research ethics committees as required in each participating hospital and jurisdiction. Patients were enrolled using priori informed consent or consent-to-continue model (also known as deferred consent) or using the opt-out model in 1 center. Because no additional patient-specific data were collected in any jurisdiction for this economic evaluation, no additional participant consent was required as per the global human research ethics committee of record for the trial.

### Health Care Resource Utilization and Unit Costs

We used patient-level data collected across all sites to capture demographic and clinical characteristics: age, sex, comorbidities, prehospital acid suppression, Acute Physiology and Chronic Health Evaluation (APACHE) II score, ICU admitting diagnosis, SARS-CoV-2 status, clinical outcomes (eg, upper gastrointestinal bleeding and death), and health care resource utilization (eg, medications, laboratory and diagnostic tests, nutrition, red blood cells and other blood products, procedures, surgeries, advanced life support strategies, and number of days in the hospital and ICU).

In the base-case analysis, unit costs for each resource item were obtained from publicly available Canadian sources. Drug unit costs were derived from provincial drug formularies in Canada^14^ and verified through consultation with hospital trial pharmacists. Unit costs for laboratory and diagnostic tests were obtained from the Schedule of Benefits for Laboratory Services,^15^ while health care professional fees were based on the Ontario Schedule of Benefits for Physician Services.^16^ Unit costs for packed red blood cells were obtained from Canadian Blood Services.^17^

Per-day ICU and ward costs were estimated using financial data submitted by hospitals across Canada to the Canadian Institute for Health Information.^18^ The daily ICU cost reflected the overall resource requirements of intensive care, including higher staffing levels, specialized personnel, complex equipment, medication use, and continuous monitoring, while the general ward cost represented routine inpatient care expenses. In the base-case analysis, we assumed that daily ICU and ward costs remained constant over the duration of stay, although this may not fully capture actual variation in costs over time.^19,20^ This approach was necessary because these were the national cost estimates available. To assess the impact of this assumption, we conducted a sensitivity analysis in which differential daily costs for ICU and ward stays were estimated based on component cost items.^16,21^ Furthermore, because these daily rates were based on aggregate data, some overlap with other itemized cost components cannot be completely excluded. We assumed that any potential overlap was likely minimal because other resource items in our analysis were specific to the REVISE trial population. We conducted 1-way sensitivity analysis by varying the daily costs by 25% higher and lower to partly address the potential overlap between itemized and aggregate costs. All unit costs are presented in eTable 1 in Supplement 1.

### Statistical Analysis

#### Primary Analysis

Total costs per patient were calculated by multiplying resource use by the corresponding unit costs and summing across all items. For patients enrolled outside of Canada, their resource use was estimated from REVISE trial data with Canadian unit costs applied because international unit costs were not collected during the trial. No discounting was applied due to the short time horizon. Incremental costs were calculated as the difference in mean per-patient costs between groups. Incremental effects were calculated as the difference in per-patient event rates between groups. In the scenario of improved effects at greater costs, we calculated the incremental cost-effectiveness ratio (ICER) in terms of the incremental cost per clinically important upper gastrointestinal bleed prevented. In the scenario of fewer bleeding events at lesser cost, we describe the intervention as dominant to the comparator and then focus on comparing the costs between treatments without calculating the ICER.

We used descriptive statistics with counts with percentages, mean with SD, and median with IQR to summarize patients’ baseline characteristics and clinical outcomes. For drugs with multiple unit costs (eg, due to different manufacturers and suppliers), we used median costs to reduce the influence of outlier pricing. Total costs were estimated and presented as mean (SD) because means and the number of patients allow for calculation of total costs, which is needed for decision-making using health economic evaluation results.

All costs were converted to 2025 US dollars (USD) considering inflation and using average currency exchange rate in 2025 (1 CAD = 0.714 USD as of August 22, 2025). All analyses were conducted according to the intention-to-treat principle. Statistical significance was set at *P* = .05 using 2-sided testing. The analyses were conducted using R version 4.4.0 (R Foundation for Statistical Computing).

#### Sensitivity Analyses

To estimate the cost-effectiveness in the US setting, we applied costs estimates in the US for key resource use items including pantoprazole, clinically important upper gastrointestinal bleeding, diagnostic tests and treatments, and ICU and hospital stay to the entire cohort (eTable 2 in Supplement 1). In addition, a series of 1-way sensitivity analyses were conducted by varying unit costs of pantoprazole, treatments for bleeding, and ICU and ward stay, reflecting variation including Australia, the US, and other European countries.^22,23^ We performed an analysis restricted to 3265 patients enrolled in 42 centers in Canada. Finally, a sensitivity analysis addressed missing resource use data using multiple imputations.^24^ In a post hoc analysis, we also excluded the top 10% of patients in terms of ICU stays, ward stays, and total cost in both groups to assess their impact with the cost saving. Probabilistic sensitivity analysis using nonparametric bootstrapping with 1000 simulations was applied to evaluate the uncertainty of the cost-effectiveness results in this patient-level, trial-based analysis.^25^

#### Subgroup Analyses

Consistent with the REVISE trial, we analyzed prespecified subgroups. Subgroups were based on (1) prehospital acid suppression use of PPI or H2-receptor antagonist (H2RA) vs no prehospital acid suppression, (2) APACHE II score (≥25 vs <25), (3) ICU admitting diagnostic category (medical vs surgical or trauma), (4) SARS-CoV-2 status (positive vs negative), and (5) sex (female vs male).^8^

## Results

### Patient Characteristics

The REVISE trial enrolled 4821 patients from 68 centers in Canada, Australia, the US, England, Saudi Arabia, Brazil, Kuwait, and Pakistan. Of all participants, 2417 were randomly assigned to the pantoprazole group and 2404 to the no pantoprazole group. The characteristics of patients included in this economic evaluation are the same as those of the trial. The mean (SD) age was 58.2 (16.4) years and 1752 patients (36.3%) were female. The mean (SD) APACHE II score was 21.7 (8.3).^3^

### Clinical Outcomes

As previously reported, clinically important upper gastrointestinal bleeding occurred in 25 patients (1.0%) receiving pantoprazole and 84 patients (3.5%) receiving placebo (hazard ratio [HR], 0.30; 95% CI, 0.19-0.47; *P* < .001). Death by 90 days occurred in 696 patients (29.1%) in the pantoprazole group and in 734 patients (30.9%) in the placebo group (HR, 0.94; 95% CI, 0.85-1.04; *P* = .25).^3^ Compared with placebo, pantoprazole was associated with fewer patient-important gastrointestinal bleeding events (36 participants [1.5%] vs 100 participants [4.2%]; *P* < .001). However, rates of pneumonia, *Clostridioides difficile,* mortality at ICU discharge, and hospital discharge were similar between groups^3^ and not considered further in this economic analysis.

### Base-Case Cost-Effectiveness

Table 1 lists the natural unit for each of the resource use items included in the analysis (eg, blood transfusions and endoscopy). The unit costs for the base-case analysis are shown in eTable 1 in Supplement 1. The mean (SD) number of days receiving pantoprazole was 8.1 (9.1) days, and the mean (SD) cost of pantoprazole was $5.10 ($5.60) per patient. In the base-case analysis including all patients receiving a treatment strategy of either pantoprazole or placebo, the group-level mean (SD) bleeding-related cost (total bleeding cost divided by all participants in each group) was $48.00 ($564.50) in the pantoprazole group vs $114.80 ($892.80) in the no pantoprazole group. The mean (SD) stay was 12.4 (11.7) days in the ICU and an additional 14.8 (28.0) days in the hospital for pantoprazole, as compared with 13.3 (13.3) days in the ICU and an additional 16.5 (42.9) days in the hospital for no pantoprazole (Table 2). For every 100 patients treated with pantoprazole, 90 (95% CI, 20-160) ICU and 170 (95% CI, 40-380) ward days were saved compared with no pantoprazole.

**Table 1.** Utilization of Medications, Tests, and Procedures

Resource	Utilization by participants, d^a^	
	Pantoprazole (n = 2417)	Placebo (n = 2404)
Medications		
Pantoprazole	19 373	0
Famotidine	43	36
Open-label pantoprazole	1414	1969
Unfractionated heparin		
Prophylactic dose	3787	3658
Therapeutic dose	1486	1573
Low molecular weight heparin		
Prophylactic dose	9896	9975
Therapeutic dose	1044	1079
Warfarin	62	50
Aspirin		
≤325 mg/d	2866	2917
>325 mg/d	15	30
Clopidogrel	313	344
New oral anticoagulants (eg, rivaroxaban)	394	405
Oral or intravenous corticosteroids	6055	6271
Anticoagulation (duration >15 d)		
Therapeutic anticoagulation	1787	2265
Prophylactic dose anticoagulation	2715	2960
Advanced life support strategies received		
Invasive mechanical ventilation	21 732	23 198
Noninvasive mechanical ventilation	898	913
Inotrope or vasopressor infusions	9735	10 054
Kidney replacement therapy		
Intermittent hemodialysis	757	733
Continuous kidney replacement therapy	1117	1543
Sustained low-efficiency dialysis	269	167
Peritoneal dialysis	25	36
Enteral or parenteral nutrition		
Enteral nutrition	22 973	24 168
Parenteral nutrition	1207	1368
Laboratory tests		
Hemoglobin	25 815	27 092
Platelets	25 444	26 708
International normalized ratio	10 232	11 109
Partial thromboplastin time	9365	10 066
Creatinine	25 360	26 697
Packed red blood cells, units	634	687
Total number of cultures, No.	6770	7082
Procedural interventions		
Upper gastrointestinal diagnostic endoscopy, No.	29	69
Therapeutic endoscopy (any), No.	16	30
Sigmoidoscopy, No.	2	2
Colonoscopy, No.	3	8
Angiogram, No.	3	6
Angio embolization and coiling, No.	3	5
Blood products		
Red blood cells, units	238	558
Fresh frozen plasma, units	76	168
Platelets, units	33	128
Cryoprecipitate, units	39	81
Medications		
Pantoprazole, No.	82	171
Famotidine, No.	0	0
Octreotide, No.	6	11
Tranexamic acid, No.	5	5
Vitamin K, No.	1	9
Hospitalization		
Intensive care unit	29 868	31 726
Ward	35 413	39 377

^a^ The total utilization of medications, diagnostic tests, and procedures among study participants by group.

**Table 2.** Base-Case Cost Effectiveness of Pantoprazole vs No Pantoprazole^a^

Outcomes	Pantoprazole (n = 2417)	No pantoprazole (n = 2404)	Incremental change (95% CI)
Clinically important upper gastrointestinal bleeding, No./total No. (%)	25/2385 (1.0)	84/2377 (3.5)	−2.5 (1.6 to 3.3)
90-d mortality, No/total No. (%)	696/2390 (29.1)	734/2379 (30.9)	−1.7 (−0.9 to 4.3)
Hospitalization			
ICU, mean (SD), d	12.4 (11.7)	13.3 (13.3)	−0.9 (−1.6 to −0.2)
Ward, mean (SD), d	14.8 (28.0)	16.5 (42.9)	−1.7 (−3.8 to 0.4)
Per patient cost, mean (SD), $^b^			
Pantoprazole	5.10 (5.60)	0	NC
Clinically important upper gastrointestinal bleeding	48.00 (564.50)	114.80 (892.80)	NC
Intensive care unit stay	41 614 (39 307)	44 441 (44 642)	NC
Ward stay	15 590 (29 754)	17 429 (45 548)	NC
Total cost, mean (SD), $	60 466 (58 546)	65 423 (75 661)	-4957 (−8777 to −1136)

Abbreviation: NC, not calculated.

^a^ Present primary outcomes, safety outcomes, and per-patient costs by group as well as the incremental cost between pantoprazole and no pantoprazole.

^b^ Per-patient costs were calculated by dividing the total cost related to the resource use item by the sample size of the treatment group.

The mean (SD) ICU costs were $41 614 ($39 307) for pantoprazole vs $44 441 ($44 642) for no pantoprazole (Table 2). The mean (SD) ward cost was $15 590 ($29 754) for pantoprazole and $17 429 ($45 548) for no pantoprazole. Mean (SD) total per-patient costs were $60 466 ($58 546) for pantoprazole vs $65 423 ($75 661) for no pantoprazole. The incremental cost per patient was −$4957 (95% CI, −$8777 to −$1136) for pantoprazole (Table 2). In terms of cost-effectiveness, pantoprazole was more effective and less costly, and thus dominant to no pantoprazole; therefore, no ICER was calculated as per our protocol.^10^

### Sensitivity Analyses

Applying US costs for pantoprazole, bleeding, and ICU and hospital stay, mean (SD) total per-patient costs were $130 179 ($123 456) for pantoprazole vs $140 770 ($153 195) for no pantoprazole (incremental cost with pantoprazole: −$10 591; 95% CI, −$18 448 to $−2735). In 1-way sensitivity analysis, using the lowest ($0.14) and highest ($8.26) pantoprazole costs (vs $0.71 at the base-case analysis), the incremental cost for pantoprazole was −$4960 and −$4890 for no pantoprazole. When the daily ICU cost varied from 25% lower to 25% higher than the cost at the base case, the incremental cost associated with pantoprazole changed from −$4250 to −$5664. If the daily cost of ward stay was 25% lower than the cost at the base case, the incremental cost with pantoprazole was −$4497 vs −$5417 when the cost was 25% higher. The incremental cost for pantoprazole varied from −$4940 to −$4974 with the change in lowest to highest cost of bleeding (eFigure 1 in Supplement 1).

Among 3265 patients in 42 Canadian centers, characteristics were similar to those of the full trial sample (eTable 3 in Supplement 1). Mean (SD) total cost per patient was $62 500 (62 283) for pantoprazole compared with $66 205 ($77 523) for no pantoprazole (incremental cost with pantoprazole: −$3704).

When excluding the top 10% of patients in terms of ICU days, ward days, and total cost to assess their impact on the cost saving, the incremental costs were −$1151, −$3388, and −$1356, respectively (eTable 4 in Supplement 1). When using differential ICU (day 1, day 2-30, and day 31 onwards) and ward (day 1-2 and day 3 onwards) daily costs, the incremental costs were −$4084 (eTable 5 in Supplement 1).

There were 15 patients with missing ICU data and 17 with missing ward data due to trial consent withdrawal in the pantoprazole group vs 11 missing ICU data and 17 missing hospital data in the placebo group. After application of multiple imputations, the incremental cost associated with pantoprazole was −$4934. In the probabilistic sensitivity analyses, in more than 99% of 1000 bootstrap simulations, pantoprazole was less costly and more effective at reducing clinically important upper gastrointestinal bleeding than no pantoprazole (Figure).

**Figure.**
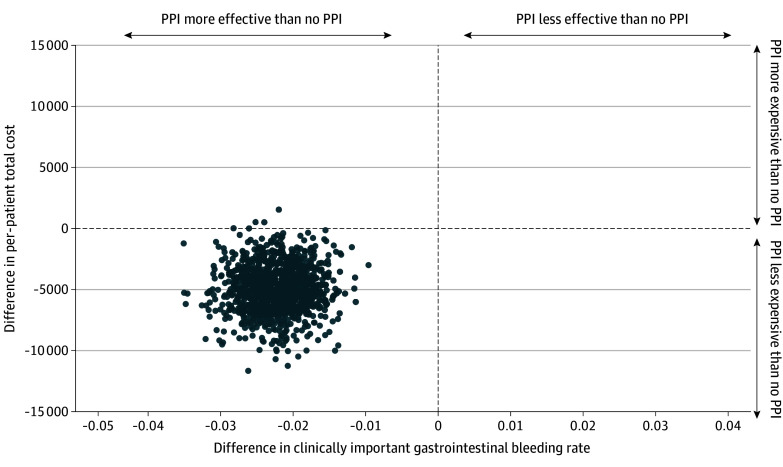
Incremental Cost-Effectiveness Between Pantoprazole and No Pantoprazole This figure presents 1000 simulations on incremental cost and incremental clinically important gastrointestinal bleeding rate between pantoprazole vs no pantoprazole. PPI indicates proton pump inhibitor.

### Subgroup Analyses

The savings using pantoprazole were consistent across all prespecified subgroups. Savings ranged from $2300 to $7300 per patient, except for those with prehospital use of acid suppression ($671; 95% CI, −$6466 to $7808) (eFigure 2 in Supplement 1).

## Discussion

In this prospective economic evaluation conducted parallel to the REVISE trial, we found that prevention of clinically important upper gastrointestinal bleeding using pantoprazole in invasively ventilated patients was cost-effective. Because the acquisition cost of pantoprazole is negligible relative to hospital-level expenditures, the reduction in resource use translated into substantial cost savings in this economic evaluation. Across multiple sensitivity and subgroup analyses, pantoprazole dominated an approach to care without stress ulcer prophylaxis. For an ICU with 1000 admissions annually, our estimates suggest potential savings of approximately $5.0 million annually for the Canadian public health care payer.

The primary factors underlying these savings were the reduction in ICU and ward length of stay, which accounted for approximately 94% of total costs, substantially augmenting savings due to fewer procedures to test or treat bleeding events in patients receiving pantoprazole. Development of clinically important upper gastrointestinal bleeding leads to emergency interventions and additional monitoring, and potentially a longer ICU admission, as found in a prior multicenter analysis^26^; avoiding this complication may result in earlier transfer to the ward, and possibly a shorter hospital stay. We also applied a broad range of unit costs to assess the robustness of findings. The magnitude of savings increased in settings with greater hospital costs. When using US costs on key items including pantoprazole, diagnostic tests and treatments for bleeding, ICU and hospital stay, the per patient saving doubled because the daily costs in ICU and hospital are greater in the US than in Canada. For other countries, savings from the use of pantoprazole can be estimated by applying alternative daily costs for variables such as the ICU or ward stay.

Published health economic evidence in this field has been limited. A decision-tree model over a 60-day time horizon for ICU patients at high risk of stress ulcer bleeding estimated total costs of $58 700 for PPIs vs $63 920 for H2RAs (2010 USD), suggesting better outcomes and modest savings with PPIs.^7^ In contrast, another model-based economic evaluation conducted over a 9-day ICU stay found that H2RAs dominated PPIs, primarily due to assumptions regarding increased risk of pneumonia and *Clostridioides difficile* infection associated with PPI use.^6^ These divergent results underscore the sensitivity of model-based analysis to underlying assumptions. Another study comparing PPI with no PPI was a cost-consequence study of 2099 Danish participants enrolled in the SUP-ICU trial^2^ which linked patient-specific data to national registry data that captured health care resource use and employment.^27^ Over 1 year, there were no differences in health care costs, resource use, or employment status between groups. Our study differs by using a trial-based analysis of a large cohort of invasively ventilated critically ill patients in 68 centers, and demonstrating the dominance of pantoprazole prophylaxis as compared with no prophylaxis, resulting in better clinical outcomes at lower cost. Additional economic evaluations with active comparators would be instructive on this topic.

### Strengths and Limitations

Strengths of this study include the preplanned design and prospective collection of detailed patient-level health care resource use alongside a large international randomized, blinded trial, enhancing internal and external validity. This study adds to the small number of randomized trials in critical care with an accompanying health economic evaluation. Of 219 economic evaluations over the last 30 years in this field, only 16% have been exclusively trial-based.^28^ Our approach was guided by a prespecified protocol and statistical analysis plan.^10^ We conducted extensive sensitivity analyses, including those using higher US costs, excluding top 10% of patients with highest costs, and differential ICU and ward daily costs, and consistently found that pantoprazole remained cost-saving.

This study also has limitations. First, the time horizon was limited to hospitalization for clinical events and 90 days for mortality, such that longer-term consequences of prophylaxis were not assessed. This report reflects unit costs of relevant resource use derived from the Canadian health care system in the base case analysis and with US and other unit costs in the sensitivity analyses. Alternative unit costs can be incorporated to approximate incremental costs between pantoprazole and no prophylaxis. To support applicability to other countries, country-specific unit costs for ICU and hospital stay can be used to estimate the cost difference between practice using pantoprazole and no pantoprazole in other settings. Jurisdictions with higher hospital costs would likely realize greater savings, as demonstrated by our sensitivity analysis using US data; the converse is also true. Quality of life data was not collected in this trial, precluding a cost-utility analysis.

## Conclusions

This economic evaluation found that the use of pantoprazole for stress ulcer prophylaxis in critically ill patients receiving invasive mechanical ventilation was more effective and less costly than no prophylaxis. These findings were due to reductions in upper gastrointestinal bleeding events and ICU and hospital stays. This study provides evidence that pantoprazole prophylaxis in invasively ventilated patients offers both clinical benefits and economic value for the health care system.

## Notes

Supplement 1. eTable 1. Unit costs for health care resource utilization in the base-case analysis eTable 2. Unit costs of key resource utilization for the U.S. scenario analysis eFigure 1. One-way sensitivity analysis eTable 3. Characteristics of Canadian patients in the REVISE trial eTable 4. Sensitivity analysis excluding top 10% of patients in terms of ICU stay, ward stay, and total costs in both groups eTable 5. Sensitivity analysis using differential ICU and ward daily costs eFigure 2. Prespecified subgroup analysis

Supplement 2. Nonauthor collaborators

Supplement 3. Data Sharing Statement
