# Supplementary material for: Cost-Effectiveness of Pantoprazole to Prevent Upper Gastrointestinal Bleeding in Mechanically Ventilated Patients
Source: JAMA Netw Open. 2025 Dec 1;8(12):e2552771. doi: 10.1001/jamanetworkopen.2025.52771 (PMC12670189; doi:10.1001/jamanetworkopen.2025.52771)
Supplement: Supplement 1. — eTable 1. Unit costs for health care resource utilization in the base-case analysis eTable 2. Unit costs of key resource utilization for the U.S. scenario analysis eFigure 1. One-way sensitivity analysis eTable 3. Characteristics of Canadian patients in the REVISE trial eTable 4. Sensitivity analysis excluding top 10% of patients in terms of ICU stay, ward stay, and total costs in both groups eTable 5. Sensitivity analysis using differential ICU and ward daily costs eFigure 2. Prespecified subgroup analysis [file jamanetwopen-e2552771-s001.pdf]

Supplemental Online Content

Xie F, Yao Y, Ma Y, et al. Cost-effectiveness of pantoprazole to prevent upper gastrointestinal bleeding in mechanically ventilated patients. *JAMA Netw. Open*. 2025;8(12):e2552771. doi:10.1001/jamanetworkopen.2025.52771

eTable 1. Unit costs for health care resource utilization in the base-case analysis

eTable 2. Unit costs of key resource utilization for the U.S. scenario analysis

eFigure 1. One-way sensitivity analysis

eTable 3. Characteristics of Canadian patients in the REVISE trial

eTable 4. Sensitivity analysis excluding top 10% of patients in terms of ICU stay, ward stay, and total costs in both groups

eTable 5. Sensitivity analysis using differential ICU and ward daily costs

eFigure 2. Prespecified subgroup analysis

This supplemental material has been provided by the authors to give readers additional information about their work.

eTable 1. Unit costs for health care resource utilization in the base-case analysis

Resources	Unit Cost, $ (range)*	Sources
**Medications, per day**		
Pantoprazole (40mg once daily)	0.64 (0.14-8.26)	Drug formularies from six provinces (Lau et al., 2022 [1])<br><br>Range: Ontario Drug Benefit Formulary (2025) [2]
Famotidine (20mg twice daily)	0.36	Ontario Drug Benefit Formulary (2025) [2]
Open-label pantoprazole (40mg once daily)	0.64	Hospital pharmacy acquisition cost provided by the trial pharmacists (2025) [3]
Unfractionated heparin		
UFH prophylactic dose (5000 IU twice daily)	5.14	
UFH therapeutic dose (25000 IU daily)	12.85	
Low molecular weight heparin (dalteparin)		
LMWH prophylactic dose (5000 IU once daily)	8.94	
LMWH therapeutic dose (10625 IU daily)	19.01	
Warfarin (5mg daily)	0.05	
Aspirin		
⩽325mg/d (81mg daily)	0.20	
>325mg/d (325mg daily)	0.81	
Clopidogrel (75mg daily)	0.17	Ontario Drug Benefit Formulary (2025) [2]
NOAC (rivaroxaban, 20mg daily)	0.70	Hospital pharmacy acquisition cost provided by the trial pharmacists (2025) [3]
Oral or IV corticosteroids (hydrocortisone, 200 mg daily)	11.12	
Anticoagulation (duration > 15 days) with UFH		
Therapeutic anticoagulation (25000 IU daily)	28.79	
Prophylactic dose anticoagulation (5000 IU once daily)	5.76	
**Advanced life support strategies received, per day**		
Mechanical ventilation (invasive or non-invasive)		Ontario Ministry of Health, Schedule of Benefits for Physician Services (2025) [4]
Day 1	131.36	
Day 2 – 30	68.86	
Day 30 onward	45.84	
Inotropes or vasopressor infusions	60.49	Hamilton Health Sciences Hospital Formulary (2020) [5]
Renal replacement therapy		Ontario Ministry of Health, Schedule of Benefits for Physician
Intermittent hemodialysis	252.90	
Continuous renal replacement therapy	271.86	

© 2025 Xie F et al. *JAMA Network Open*.

Sustained low-efficiency dialysis	226.52	Services (2025) [4]
Peritoneal dialysis	169.50	
**Enteral, or parenteral nutrition, per day**		
Enteral nutrition	65.17	Ontario Ministry of Health, Schedule of Benefits for Physician Services (2025) [4]
Parenteral nutrition	19.39	
**Laboratory Tests, per day**		
Hemoglobin	2.84	Ontario Ministry of Health, Schedule of Benefits for Laboratory Services (2025) [6]
Platelet count	2.84	
International normalized ratio	1.90	
Partial thromboplastin time	1.90	
Creatinine	9.60	
**Total number of cultures, No**	7.92	Ontario Ministry of Health, Schedule of Benefits for Laboratory Services (2025) [6]
**Procedural Intervention, No****		
Upper gastrointestinal diagnostic endoscopy (procedure, sedation and consultation)	215.21	Ontario Ministry of Health, Schedule of Benefits for Physician Services (2025) [4]
Therapeutic endoscopy (procedure, sedation and consultation)	285.22	
Management of bleeding (any technique: injection, clipping, banding, diathermy, laser, hemospray, Blakemore tube, etc.)	33.06	
Sigmoidoscopy (procedure, consultation plus ICU premium)	252.83	
Colonoscopy (procedure, consultation plus ICU premium)	248.73	
Angiogram (consultation with radiologist)	119.63	
Angio embolization/coiling (consultation with radiologist)	119.63	
**Treatments of bleeding events****		
Transfusion, per unit		Blood Component Cost Per Unit Summary, Canadian Blood Services (2024) [7]
Red blood cells	322.87	
Fresh frozen plasma	91.46	
Platelets	287.99	
Cryoprecipitate	111.96	
Treatment Medication, per day		Drug formularies from six provinces (Lau et al., 2022 [1])
Pantoprazole (40mg twice daily)	1.28	
Famotidine (40mg twice daily)	0.72	
Octreotide (1.2mg infusion daily)	94.11	
Tranexamic acid (3000mg once daily)	808.07	
Vitamin K (10mg once daily)	11.89	Healthy Planet Canada (2025) [8]

© 2025 Xie F et al. *JAMA Network Open*.

**Hospitalization, per day**		
ICU	3,367.50 (2,525.63-4,209.38)	Canadian Institute for Health Information. Care in Canadian ICUs. CIHI (2016) [9]
Ward	1,064.06 (798.05-1,330.08)	

Legend for eTable 1: In this table, we show the unit costs for health care resource utilization as used in the base-case analysis of this economic evaluation.
*The lowest and highest costs for key resource items were listed in parentheses and used in one-way sensitivity analyses, where each cost was varied individually across this range while holding all other costs constant.

**Considering the sum of endoscopy (diagnostic or therapeutic), angiography (with or without embolization) or surgery, 2 units of packed red blood cells and pantoprazole 40mg twice daily was $81.31. This does not incorporate other costs of additional tests, length of stay in the ICU or hospital. The range (±25%), $60.99 – $101.64, was used in the one-way sensitivity analysis.

All costs were adjusted to 2025 values using the Canadian Consumer Price Index (CPI). All costs were converted to U.S. dollars (USD) using an average 2025 exchange rate of 1 CAD = 0.714 USD (as of August 2025).
UFH=unfractionated heparin; LMWH=low molecular weight heparin; NOAC=new oral anticoagulants; ICU=intensive care unit.

References

[1] Lau VI, Xie F, Fowler RA et al. Health economic evaluation alongside the Probiotics to Prevent Severe Pneumonia and Endotracheal Colonization Trial (E-PROSPECT): a cost-effectiveness analysis. Can J Anesth/J Can Anesth 2022;69:1515–1526.
[2] Ontario Ministry of Health. Ontario Drug Benefit Formulary. https://www.formulary.health.gov.on.ca/formulary/
[3] Hospital pharmacy acquisition cost provided by the trial pharmacists. Internal data; 2025.
[4] Ontario Ministry of Health. Schedule of Benefits for Physician Services [PDF]. Ontario; 2025. https://www.ontario.ca/files/2025-03/moh-schedule-benefit-2025-03-19.pdf
[5] Hamilton Health Sciences. Hospital Formulary. Hamilton, ON; 2020.
[6] Ontario Ministry of Health. Schedule of Benefits for Laboratory Services [PDF]. Ontario; 2025. https://www.ontario.ca/files/2025-03/moh-ohip-schedule-of-benefits-laboratory-services-2025-03-03.pdf
[7] Canadian Blood Services. 2023–2024 Blood Component Cost per Unit Summary [PDF]. Ottawa, ON: Canadian Blood Services; 2024. https://www.blood.ca/en/hospital-services/products/component-types
[8] Healthy Planet Canada. Vitamins & Supplements.2025. https://www.healthyplanetcanada.com/vitamins-supplements.html.
[9] Canadian Institute for Health Information. Care in Canadian ICUs [PDF]. Ottawa, ON: CIHI; 2016. https://www.cihi.ca/sites/default/files/document/care-canadian-icu-report-en.pdf

© 2025 Xie F et al. *JAMA Network Open.*

eTable 2. Unit costs of key resource utilization for the U.S. scenario analysis

Resources	Mean	Sources for the US Scenario
Pantoprazole (40mg IV once daily)	$2.41	Hernandez et al. 2023 [1]
Bleeding	$20,299.52	Hammond et al. 2017 [2]
ICU day	$6,407.00	Halpern et al. 2016 [3]
Ward day	$1,729.01	Donahoe et al. 2012 [4]
Mechanical ventilation (invasive or non-invasive)	$2,361.00	Zilberberg et al. 2008 [5]
Renal replacement therapy		
Intermittent Hemodialysis	$280.92	Ethgen et al. 2023 [6]
Continuous Renal Replacement Therapy	$897.29	Ethgen et al. 2023 [6]
Sustained Low-Efficiency Dialysis	$1,221.00	Ahmed et al. 2009 [7]
Peritoneal dialysis	$307.08	Kaplan et al. 2022 [8]

Legend for eTable 2: This table presents the U.S. values for key resource utilization that were used in sensitivity analyses. All prices were converted to 2025 USD using the U.S. CPI for Medical Care published by the U.S. Bureau of Labor Statistic.

References

[1] Hernandez IA, Morell J, Mulcahy L, Luzardo D. Comparison Between Pantoprazole Intermittent Dosing and Continuous Infusion in Suspected Upper Gastrointestinal Bleeding Prior to Endoscopy: Impact of a Pharmacist-Driven Protocol to Reduce Utilization of Pantoprazole Continuous Infusion. Cureus. 2023;15(10):e48056.

[2] Hammond DA, Kathe N, Shah A, Martin BC. Cost-Effectiveness of Histamine2 Receptor Antagonists Versus Proton Pump Inhibitors for Stress Ulcer Prophylaxis in Critically Ill Patients. Pharmacotherapy. 2017;37(1):43-53.

[3] Halpern NA, Goldman DA, Tan KS, Pastores SM. Trends in Critical Care Beds and Use Among Population Groups and Medicare and Medicaid Beneficiaries in the United States: 2000-2010. Crit Care Med. 2016;44(8):1490-1499.

[4] Donahoe MP. Current venues of care and related costs for the chronically critically ill. Respir Care. 2012;57(6):867-888.

[5] Zilberberg MD, Luippold RS, Sulsky S, Shorr AF. Prolonged acute mechanical ventilation, hospital resource utilization, and mortality in the United States. Crit Care Med. 2008;36(3):724-30.

[6] Ethgen O, Murugan R, Echeverri J, Blackowicz M, Harenski K, Ostermann M. Economic Analysis of Renal Replacement Therapy Modality in Acute Kidney Injury Patients With Fluid Overload. Crit Care Explor. 2023;5(6):e0921.

[7] Ahmed Z, Gilibert S, Krevolin L. Cost analysis of continuous renal replacement and extended hemodialysis. Dial. Transplant 2009;38: 500-503.

[8] Kaplan JM, Niu J, Ho V, Winkelmayer WC, Erickson KF. A Comparison of US Medicare Expenditures for Hemodialysis and Peritoneal Dialysis. J Am Soc Nephrol. 2022;33(11):2059-2070

© 2025 Xie F et al. *JAMA Network Open.*

eFigure 1. One-way sensitivity analysis

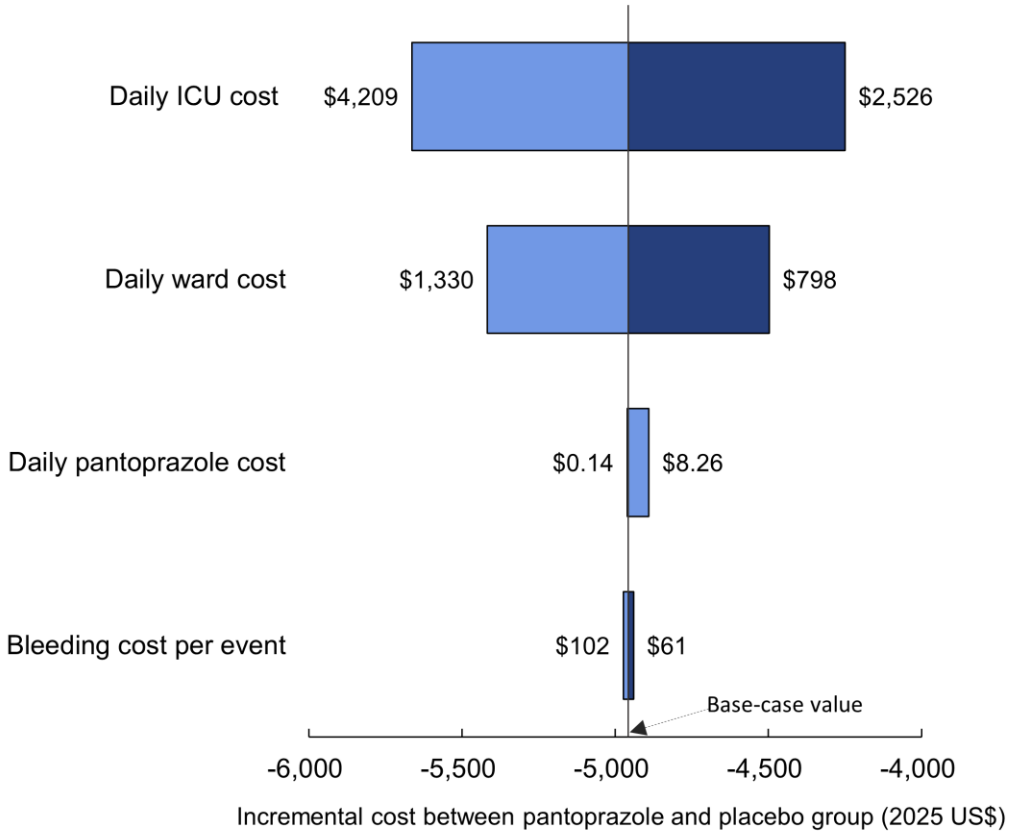

Legend for eFigure 1: This figure presents one-way sensitivity analyses by varying the cost of pantoprazole, gastrointestinal bleeding, ICU and ward stays (the dollar amount at both ends of each bar was the lowest and highest costs used).

© 2025 Xie F et al. *JAMA Network Open*.

eTable 3. Characteristics of Canadian patients in the REVISE trial

Characteristic	Pantoprazole	Placebo
Number of participants	1638	1627
Age in years (mean ± standard deviation)	59.3 ± 16.0	59.3 ± 16.3
APACHE II score	22.1 ± 8.4	22.1 ± 8.2
Sex - no. (%)		
Female	584 (35.7%)	617 (37.9%)
Male	1054 (64.3%)	1010 (62.1%)
Patient status - no. (%)		
Medical	1259 (76.9%)	1258 (77.3%)
Surgical	166 (10.1%)	182 (11.2%)
Trauma	213 (13.0%)	187 (11.5%)
Corticosteroid ≥1 week pre-randomization - no. (%)	616 (37.6%)	609 (37.4%)
Type of life support - no. (%)		
Invasive mechanical ventilation	1638 (100.0%)	1627 (100.0%)
Non-invasive mechanical ventilation	209 (12.8%)	218 (13.4%)
Inotropes or vasopressor infusions	1245 (76.0%)	1245 (76.5%)
Renal replacement therapy	192 (11.7%)	195 (12.0%)

Legend for eTable 3: In this table, we present characteristics of patients in Canadian sites reflecting one of the sensitivity analyses.

APACHE II score = Acute Physiology and Chronic Health Evaluation II Score

© 2025 Xie F et al. *JAMA Network Open*.

eTable 4. Sensitivity analysis excluding top 10% of patients in terms of ICU stay, ward stay, and total costs in both **groups**

	**Pantoprazole**	**No pantoprazole**	**Difference**
**Excluding top 10% with longest ICU stay**			
**n**	2,189	2,147	
**Total cost**	$48,648 (36,055)	$49,799 (38,683)	-$1,151
**Excluding top 10% with longest ward stay**			
**n**	2,172	2,152	
**Total cost**	$49,864 (39,154)	$53,252 (44,139)	-$3,388
**Excluding top 10% with highest total cost**			
**n**	2,187	2,152	
**Total cost**	$45,921 (28,371)	$47,277 (29,185)	-$1,356

© 2025 Xie F et al. *JAMA Network Open*.

eTable 5. Sensitivity analysis using differential ICU and ward daily costs

	Pantoprazole	No pantoprazole	Difference
**n**	2417	2404	
**ICU days**	12.4 (11.7)	13.3 (13.3)	
**ICU cost**	$33,693 (31,434)	$35,942 (35,619)	
**Ward days**	14.8 (28)	16.5 (42.9)	
**Ward costs**	$13,125 (25,005)	$14,670 (38,273)	
**Total cost**	$50,080 (48,231)	$54,164 (62,443)	-$4,084

ICU daily costs: $2,838 (day 1), $2,724 (day 2-30); and $2,642 (day 31 onwards).
Ward daily cost: $913 (day 1-2), and $894 (day 3 onwards).

References:

[1] Ontario Ministry of Health. Schedule of Benefits for Physician Services [PDF]. Ontario; 2024. https://www.ontario.ca/files/2025-03/moh-schedule-benefit-2025-03-19.pdf

[2] Tarride JE, Blackhouse G, Rochwerg B, et al. Cost-Effectiveness of In-Bed Cycling and Routine Physiotherapy for Patients Receiving Mechanical Ventilation. JAMA Netw Open. Sep 2 2025;8(9):e2529399.

eFigure 2. Prespecified subgroup analysis

Subgroup	N	Pantoprazole Mean (SD)	N	Placebo Mean (SD)	Incremental cost Mean (95% CI)
**Use of acid suppression before hospitalization**					
No	1847	60206 (58116)	1854	66842 (79260)	-6636 (-11113, -2160)
Yes	570	61308 (59963)	550	60637 (61833)	671 (-6466, 7808)
**APACHE II score**					
<25	1595	60842 (55776)	1600	64583 (64824)	-3741 (-7934, 452)
≥25	822	59736 (63607)	804	67094 (93590)	-7358 (-15153, 437)
**Diagnosis on ICU admission**					
Medical	1753	59730 (61080)	1767	64878 (81046)	-5148 (-9887, -409)
Surgical or trauma	664	62407 (51251)	637	66933 (58199)	-4525 (-10494, 1443)
**SARS-CoV-2 status**					
Active infection	184	78136 (85285)	177	80440 (76741)	-2305 (-19028, 14419)
No active infection	2233	59010 (55552)	2227	64229 (75464)	-5219 (-9109, -1329)
**Sex**					
Female	883	59697 (55652)	870	64544 (78111)	-4846 (-11204, 1511)
Male	1534	60908 (60162)	1534	65921 (74256)	-5013 (-9796, -231)

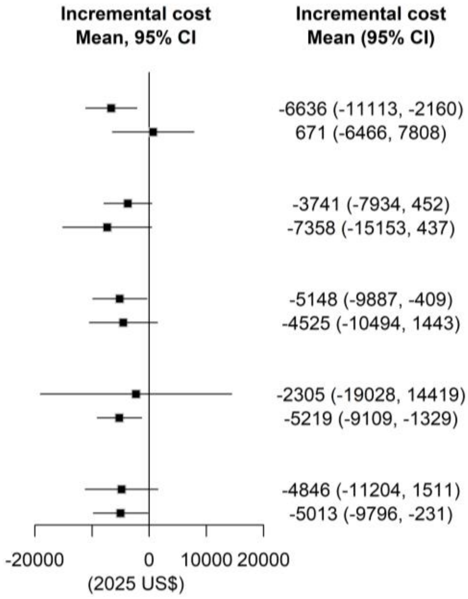

Legend for eFigure 2: This figure shows subgroup analyses on five prespecified subgroup analyses in the REVISE trial.

APACHE II Score=Acute Physiology and Chronic Health Evaluation II score;
ICU=intensive care unit; SARS-CoV-2=Severe acute respiratory syndrome coronavirus 2

© 2025 Xie F et al. *JAMA Network Open*.
