# Supplementary material for: Cost-Effectiveness of Pantoprazole to Prevent Upper Gastrointestinal Bleeding in Mechanically Ventilated Patients
Source: JAMA Netw Open. 2025 Dec 1;8(12):e2552771. doi: 10.1001/jamanetworkopen.2025.52771 (PMC12670189; doi:10.1001/jamanetworkopen.2025.52771)
Supplement: Supplement 2. — Nonauthor collaborators [file jamanetwopen-e2552771-s002.pdf]

Supplemental Online Content: Nonauthor Collaborators
*First name, last name, and suffix (if applicable) are required and will appear in PubMed.

***Group Name(s):** E-REVISE Collaborators and Canadian Critical Care Trials Group

***First Name and Middle Initial(s)**	***Last Name**	***Suffix (eg, Jr, III)**	Academic Degrees	Institution	Location (city, state/province, country)	Role or Contribution, eg, chair, principal investigator	Group (if more than 1 Group listed in the byline) and/or Subgroup (eg, Steering Committee)
Francois	Lauzier		MD, MSc	Université Laval	Quebec City, Quebec, Canada	Investigator, Adjudicator	Canadian Steering Committee
Lois	Saunders		N/A	St. Joseph's Healthcare Hamilton	Hamilton, Ontario, Canada	Global Data Manager	McMaster University Methods Center
Miranda	Hardie		M.Clin.Epi	The George Institute	Sydney, Australia	Project Manager, Australia	Methods Center, The George Institute
Waleed	Alhazzani		MD, MSc	McMaster University	Hamilton, Ontario, Canada	Investigator	International Steering Committee
John	Muscedere		MD	Queen’s University	Kingston, Ontario, Canada	Investigator	Canadian Steering Committee
Shane	English		MD, MSc	University of Ottawa	Ottawa, Ontario, Canada	Investigator	Canadian Steering Committee
Serena	Knowles		RN, PhD	The George Institute	Sydney, Australia	Project Manager, Australia	Methods Center, The George Institute
Naomi	Hammond		RN, PhD	The George Institute	Sydney, Australia	Investigator	Methods Center, The George Institute
Kathleen M.	Byrne		RN	Royal Melbourne Hospital	Melbourne, Australia	Research Coordinator	
Marianne	Chapman		MD, PhD	University of Adelaide	Adelaide, Australia	Investigator	Australian Steering Committee
Balasubramanian	Venkatesh		MD	The George Institute	Sydney, Australia	Investigator	Methods Center, The George Institute
Paul	Young		MD, PhD	Wellington Hospital	Wellington, New Zealand	Investigator	International Steering Committee
Dorrilyn	Rajbhandari		RN	The George Institute	Sydney, Australia	Investigator	Methods Center, The George Institute
Abdulrahman	Al-Fares		MD	Al-Amiri Hospital	Kuwait City, Kuwait	Investigator, Adjudicator	International Steering Committee
Gilmar	Reis		MD, PhD	Pontifical Catholic University	Belo Horizonte, Brazil	Investigator	International Steering Committee
Mobeen	Iqbal		MD	Maroof International Hospital	Islamabad, Pakistan	Investigator	International Steering Committee
Richard	Hall		MD, PhD	Dalhousie University	Halifax, Nova Scotia, Canada	Investigator	Canadian Steering Committee
Maureen	Meade		MD, MSc	McMaster University	Hamilton, Ontario, Canada	Investigator	
Lori	Hand		BSc, RRT,CCRA	McMaster University	Hamilton, Ontario, Canada	Research Coordinator	
Erick	Duan		MD, MSc	McMaster University	Hamilton, Ontario, Canada	Investigator	
France	Clarke		RRT	McMaster University	Hamilton, Ontario, Canada	Research Coordinator	
Joanna	Dionne		MD, PhD	McMaster University	Hamilton, Ontario, Canada	Investigator, Adjudicator	
Jennifer LY	Tsang		MD, PhD	McMaster University	Hamilton, Ontario, Canada	Investigator	
Timothy	Karachi		MD	McMaster University	Hamilton, Ontario, Canada	Investigator	
François	Lamontagne		MD, MSc	Universitaire de Sherbrooke	Sherbrooke, Quebec, Canada	Investigator	
Frederick	D'Aragon		MD, PhD	Universitaire de Sherbrooke	Sherbrooke, Quebec, Canada	Investigator	
Charles	St. Arnaud		MD	Universitaire de Sherbrooke	Sherbrooke, Quebec, Canada	Investigator	
Brenda	Reeve		MD	Brantford General Hospital	Brantford, Ontario, Canada	Investigator	
Daniel	Niven		MD, PhD	University of Calgary	Calgary, Alberta, Canada	Investigator	
Gloria	Vazquez-Grande		MD, PhD	University of Manitoba	Winnipeg, Manitoba, Canada	Investigator	
Ryan	Zarychanski		MD, MSc	University of Manitoba	Winnipeg, Manitoba, Canada	Investigator	
Daniel	Ovakim		MD, MSc	Victoria General Hospital	Victoria, British Columbia, Canada	Investigator	
Gordon	Wood		MD	Victoria General Hospital	Victoria, British Columbia, Canada	Investigator	
Karen E.A.	Burns		MD, MSc	University of Toronto	Toronto, Ontario, Canada	Investigator	
Alberto	Goffi		MD, PhD	University of Toronto	Toronto, Ontario, Canada	Investigator	
M. Elizabeth	Wilcox		MD, PhD	University of Toronto	Toronto, Ontario, Canada	Investigator, Adjudicator	
William	Henderson		MD, PhD	University of British Columbia	Vancouver, British Columbia, Canada	Investigator	
David	Forrest		MD, MSc	Nanaimo Regional General Hospital	Nanaimo, British Columbia, Canada	Investigator	
Neill	Adhikari		MD, MSc	Sunnybrook Health Sciences Center	Toronto, Ontario, Canada	Investigator	
Ian	Ball		MD, MSc	Western University	London, Ontario, Canada	Investigator	
Tina	Mele		MD, PhD	Western University	London, Ontario, Canada	Investigator	
Alexandra	Binnie		MD, DPhil	William Osler Hospital	Brampton, Ontario, Canada	Investigator	
Sebastien	Trop		MD, PhD	William Osler Hospital	Brampton, Ontario, Canada	Investigator	

Supplemental Online Content: Nonauthor Collaborators
*First name, last name, and suffix (if applicable) are required and will appear in PubMed.

***First Name and Middle Initial(s)**	***Last Name**	***Suffix (eg, Jr, III)**	Academic Degrees	Institution	Location (city, state/province, country)	Role or Contribution, eg, chair, principal investigator	Group (if more than 1 Group listed in the byline) and/or Subgroup (eg, Steering Committee)
Sangeeta	Mehta		MD	Mount Sinai Hospital	Toronto, Ontario, Canada	Investigator	
Ingrid	Morgan		MD, MSc	Cambridge Memorial Hospital	Cambridge, Ontario, Canada	Investigator	
Osama	Loubani		MD	Dalhousie University	Halifax, Nova Scotia, Canada	Investigator	
Meredith	Vanstone		PhD	McMaster University	Hamilton, Ontario, Canada	Investigator	
Kristen	Fiest		PhD	University of Calgary	Calgary, Alberta, Canada	Investigator	
Emmanuel	Charbonney		MD, PhD	University of Montréal	Notre Dame, Quebec, Canada	Investigator	
Alexandros	Cavayas		MD, MSc	Sacre Coeur Hospital	Montreal, Quebec, Canada	Investigator	
Oleksa	Rewa		MD, MSc	University of Alberta	Edmonton, Alberta, Canada	Investigator	
Arnold S.	Kristof		MD, PhD	McGill University	Montreal, Quebec, Canada	Investigator	
Eric	Sy		MD, MPH	University of Saskatchewan	Regina, Saskatchewan, Canada	Investigator	
Brittany	Dennis		MD, PhD	University of British Columbia	Vancouver, British Columbia, Canada	Investigator	
Steven	Reynolds		MD	Royal Columbia Hospital	New Westminister, British Columbia, Cana	Investigator	
François	Marquis		MD, MA	Hôpital Maisonneuve Rosemont	Montreal, Quebec, Canada	Investigator	
François	Lellouche		MD, PhD	Université Laval	Quebec City, Quebec, Canada	Investigator	
Adam	Rahman		MD	Windsor Regional Hospital	Windsor, Ontario, Canada	Investigator	
Paul	Hosek		MD	Grand River Hospital	Kitchener, Ontario, Canada	Investigator	
Robert	Cirone		MD	Unity Health Network	Toronto, Ontario, Canada	Investigator	
Mark	Tutschka		MD	Saint John Regional Hospital	Saint John, New Brunswick, Canada	Investigator	
Laurent	Billot		PhD	The George Institute	Sydney, Australia	Biostatistician	Australian Steering Committee
Lehana	Thabane		PhD	McMaster University	Hamilton, Ontario, Canada	Biostatistician	International Steering Committee
Simon	Finfer		MBBS	The George Institute	Sydney, Australia	Chief Australian Investigator, Adjudicator	International Steering Committee
