# Supplementary material for: Cost-Effectiveness of Pantoprazole to Prevent Upper Gastrointestinal Bleeding in Mechanically Ventilated Patients
Source: JAMA Netw Open. 2025 Dec 1;8(12):e2552771. doi: 10.1001/jamanetworkopen.2025.52771 (PMC12670189; doi:10.1001/jamanetworkopen.2025.52771)
Supplement: Supplement 3. — Data Sharing Statement [file jamanetwopen-e2552771-s003.pdf]

Data Sharing Statement

Xie. Cost-Effectiveness of Pantoprazole to Prevent Upper Gastrointestinal Bleeding in Mechanically Ventilated Patients. *JAMA Netw Open*. Published December 01, 2025. doi:10.1001/jamanetworkopen.2025.52771

Data
Data available: No
